# Filling the Gaps in Adolescent Care and School Health Policy-Tackling Health Disparities through Sports Medicine Integration

**DOI:** 10.3390/healthcare6040132

**Published:** 2018-11-13

**Authors:** Kemba Noel-London, Anthony Breitbach, Rhonda Belue

**Affiliations:** 1 College for Public Health and Social Justice Department of Health Management and Policy, Saint Louis University, St. Louis, MO 63104, USA; rhonda.belue@slu.edu; 2 Doisy College of Health Sciences, Department of Physical Therapy and Athletic Training, Saint Louis University, St. Louis, MO 63104, USA; anthony.breitbach@health.slu.edu

**Keywords:** School-Based Health Centres, adolescent healthcare, health disparities, sports medicine, Athletic Training, community health, social determinants

## Abstract

The School-Based Health Centre (SBHC) model of healthcare delivery in community health is designed to address the unique needs of adolescents. Through a collaborative interprofessional approach, they aim to provide comprehensive care with the goal of reducing health disparities in underserved, at-risk adolescents. Integration of sports medicine health professionals is a novel approach to increasing available services, as well as patient utilization, while addressing multiple public health issues, including lack of athletic training services for youth athletes.

## 1. Introduction

Adolescence is often thought of as an optimal time to lay the foundation for good health behaviours that can carry into adulthood. Acquiring positive health habits such as physical activity, at this stage of development is critical to preventing the onset of chronic diseases such as diabetes and obesity in adulthood [1,2]. Traditional models of healthcare in school systems, often manned by nurses, were designed to address communicable diseases. They were not designed to manage the chronic diseases that are becoming more common among young people [3]. These diseases often have multifaceted influencers, independent of biologic pathways. Integrating healthcare practice beyond traditional systems is the driver behind this proposed model of school-based healthcare delivery. Inclusion of healthcare professions such as Athletic Training (AT), though overlooked, may be a useful component in the reduction of non-emergent emergency department visits and reduction of healthcare expenditure; both of which align with the goals and desired outcomes of SBHCs. Therefore, it is prudent to consider its utility to the broader landscape of healthcare, as well as its potential to impact the widespread inequalities that impact patient’s health outcomes.

Throughout this commentary, we will explore School-Based Health Centres, their role in community healthcare and addressing health inequities in youth and the value that sports medicine health professionals such as athletic trainers, can add to adolescent healthcare. Our aim is to present and discuss the merits of a model that builds on traditional school health clinics and expands the services offered, by integrating sports medicine health professionals.

### 1.1. School-Based Health Centres

The School-Based Health Centre (SBHC) model of healthcare is targeted to address the unique needs of often underserved and at-risk adolescent populations [4]. It is a collaborative interprofessional care model that provides comprehensive care via a partnership between high schools or middle schools and local healthcare institution. They offer a range of services providing comprehensive care and often function as the primary healthcare provider for students who may have significant barriers to access [5,6]. SBHCs can greatly impact multiple facets of an adolescent’s academic and emotional development. They have been shown to reduce healthcare disparity gaps and advance health equities in low socioeconomic status and ethnically diverse populations [4,7]. Typically, services of an SBHC can include reproductive health services, nutrition services, health promotion, health education, case management services, health education and mental health services in an interprofessional approach to deliver holistic care and improve outcomes. SBHCs are primarily staffed by a nurse practitioner or physician assistant in collaboration with a licensed physician as the primary care provider (PCP). The physician’s role is more administrative with minimal patient contact hours. Clinical support to PCPs is done via a registered or licensed practical nurse with assistance from a medical assistant, with dental services and social services also being offered. The SBHCs use three primary staffing models. The **Primary Care Model**, is the most prevalent and other models use this as the building block for other frameworks. The **Primary Care Mental Health Model** adds a licensed clinical social worker, psychologist or substance abuse counsellor to the aforementioned model. The most comprehensive model, **Primary Care Mental Health Plus** includes other health professionals, typically a health educator, case manager or nutritionist along with the PCP and mental health provider [8].

In the United States (US), SBHCs have also been shown to be cost beneficial to the Medicaid program [9]. Management of childhood asthma through a nationwide SBHC program has an estimated total savings of $23.13 billion [3]. The management of childhood asthma and diabetes through SBHC results in an estimated savings of $970/child [3]. Adolescent users of SBHCs are also more likely to complete immunization series that required multiple doses when compared to non-users [10]. There is also emerging evidence that the SBHC model positively impacts educational outcomes such as absenteeism and graduation rates [5,11,12,13]. SBHCs in the US are primarily found in schools where 50% of students are eligible for free or reduced price lunch [14,15]. It may be of note for policy advancement that there is the need to evaluate the utility of SBHCs in high-income minority populations, given that health disparities persist in these populations regardless of the protective population effect of high socioeconomic status (SES).

### 1.2. Socioeconomic Status and Healthcare

Through the socioeconomic gradient, which can be measured by a person’s income, occupation, or highest level of education achieved, we understand that as an individual’s socioeconomic status changes, health outcomes can also change [16,17,18,19]. In the framework proposed by Williams and Mohammed, currently, in many countries, race combined with socioeconomic status (SES) and other demographic factors (gender, age etc.) combine to determine and individual’s ‘social status’. In combination with basic causes such as biology and geographical origins, societal institutions and racism, these impact health through various proximal pathways and influence behavioural, psychological and physiological responses [20]. Nonetheless, education is still one of the strongest and most consistent predictors of health [21,22,23,24,25,26]. The impact of the reverse causality nature of health and education is well established in the literature [22,23,24,25,26]. Through ecological theory we understand that adolescents can be influenced by home, school and community [27]. Attempting to improve adolescent health outcomes through a single pronged approach, is not the way to move the needle. Incorporating health and education, while embracing the role of the communities that they are living and matriculating in, is the approach that is the driver behind the improved coordinated school health model: Whole School, Whole Community, Whole Child model (WSCC) [28,29,30,31]. The recognized need for an expanded model to school health intertwines public health and education to achieve the common goals of both sectors. SBHCs are an option for policy makers to consider when trying to develop policies and strategies that fit within the WSCC.

The estimated economic cost of persistent social disparities and resulting health disparities, is staggering. Cost savings were estimated to be $230 billion for 2003–2006; should these disparities be addressed in the US healthcare system [32]? Substantial savings, an estimated 1 billion, in inpatient hospital charges can be saved by eliminating racial and ethnic differences in mental healthcare alone [33].

Minority adolescents with low SESs have been shown to have low immunization rates, poor management of chronic diseases and limited access to mental and behavioural health services [10,34,35]. We acknowledge that this does not always hold true for high-income minorities [36,37,38] and while this trend may not be evident in higher SES adolescents, it is important to note the protective effect of high SES does not always exist for minority adolescents when examining various health outcomes [39,40]. Failure to address these very costly disparities in the young, will undoubtedly increase societal and economic costs.

### 1.3. Sports Medicine Integration

Incorporating sports medicine health professionals such as Athletic Trainers (ATs) in the SBHC model fits within the goals of the implementing the WSCC model at schools [41]. ATs are defined as:

“*Healthcare professionals who render service or treatment, under the direction of or in collaboration with a physician, in accordance with their education and training and the states’ statutes, rules and regulations. As a part of the healthcare team, services provided by ATs include injury and illness prevention, wellness promotion and education, emergent care, examination and clinical diagnosis, therapeutic intervention, and rehabilitation of injuries and medical conditions. Athletic training is recognized by the American Medical Association (AMA) as a healthcare profession.*”

[42]

It is widely recommended that children and adolescents engage in regular physical activity to promote wellness and improved health outcomes [2]. Participation in extracurricular activities is sometimes used as a method to achieve the advised 60 min of moderate physical activity per day [43]. Thirty eight million youth participate in organised sport nationwide but approximately 1/3 of public schools have access to a full time AT which is a startling figure given that 1.4 million high school sport related injuries occur every year [44,45]. Youth deaths occurring from heart conditions, head injuries or exertional heat stroke are preventable if the right equipment, protocol and health professional is in place [44]. Involvement in any sport comes with inherent risk. The American Academy of Family Physicians recommends that schools that provide interscholastic sports, have an AT be present and be part of the athletic care team [46]. Given the vulnerability of the adolescent population to concussion sequalae [47,48,49], the American Academy of Paediatrics recommends that AT be available on the side-line at football practices and games, as evidence shows that their presence can impact injury rates for athletes [50]. Research has shown an AT’s diagnostic accuracy regarding orthopaedic injury and referral to be on-par with that of physicians [51]. Adolescents also may have difficulty with the “return to learn” process post-concussive injury, if a concussion is not managed properly or there are no policies in place to establish management guidelines [52,53]. ATs are well equipped to evaluate, manage and be the bridge between return to sport and return to learn, ensuring that an adolescent’s academic life, both short term and long term, is not negatively impacted by their health [54,55]. Utilising the Nationwide Emergency Department Sample dataset, Nalliah et al. found that in 2008, there were 432,609 sport related emergency department visits by youth between the age of 13 to 19 years old. The resultant total cost of these visits were $447.4million [56]. The bulk of these injuries were contusions, sprains and strains, typically non-emergent or life threatening injuries [56]. In 2013, 24% of sport related emergency department visits in the US were sprains [57]. This is a staggering cost and burden that should and can be reduced. These figures shed light on an overlooked secondary public health problem that is direct opposition to achieving the goals of the WSCC. The broad skillset of ATs can be very advantageous in the population served by SBHCs, however, despite the increasing number of SBHCs operating in 49 of the 50 states and District of Columbia, rehabilitative, sports and physical medicine are not services that are typically provided through SBHCs. The Mercy Clinic in St. Louis MO is the only SBHC in the nation that has the services of a Certified Athletic Trainer directly. Here, the AT was provided by the neighbouring university via a graduate assistant program. In addition to providing AT services at the SBHC and for the school’s athletic programs, the AT also serves as an adjunct instructor in the Department of Physical Therapy and Athletic Training.

## 2. Challenges and Recommendations

The American Academy of Family Physicians recommends that policies are created to allow for the utilization of an AT to enhance the well-being of student athletes and encourages boards of education and departments of health to work with schools to develop and implement said policies [46]. However, athletic directors, who want to hire ATs, often face notable barriers to doing so as hiring and budgeting decisions are made often by school boards [58]. Incorporating ATs into the high school community through an SBHC may be a novel way to address obstacles faced by athletic directors and school boards while tackling multiple public health concerns. ATs can be provided via community partnerships with Universities, colleges or non-profit organisations like the method utilised in St. Louis. Additionally, ATs can be provided by SBHCs and their partners if backed by hospital systems who have established sports medicine programs. The SBHC would provide common setting for the AT to practice and improve communication by reducing medical information silos, in addition to promote interprofessional collaborative practice. Additionally, providing an AT to the SBHC through community or educational partnerships, may offset the financial hiring cost for schools and school districts. This framework would also promote continuity of care in all facets of the adolescent’s school-based healthcare experience.

Regulation of the athletic training profession is also not standardized and varies from state to state. This may pose a limitation of the adaption of this model in some states, depending on laws. The profession, while commonly known and found in college and secondary school athletic settings, is expanding to less commonly thought of settings [59]. According to the National Athletic Training Association, 17% of all ATs can be found in the clinic or hospital setting [59]. This shows the wide applicability of AT skills is being embraced and utilised in the wider healthcare industry. Additionally, studies have shown that the addition of ATs to physician practices resulted in increases in patient throughput of 15–30%, with a corresponding increase in income [60,61,62,63]. Pecha et al. found that utilising ATs as physician extenders in clinics, increased revenue by $200 to $1200 per day [60]. While, there are limitations regarding billing for Medicare/Medicaid patients, an athletic trainer can provide valuable expertise to a healthcare team as these payors move toward bundled and value-based reimbursement models [64]. One study has shown that as a part of the healthcare team, services provided by ATs include injury and illness prevention, wellness promotion and education, emergent care, examination and clinical diagnosis, therapeutic intervention, and rehabilitation of injuries and medical conditions.

Advanced rehabilitative and preventive services for the active youth population can be provided through the SBHC with an AT on staff, a service that is not currently delivered, but is key in the longevity and safety of athletes In addition to injury rehabilitation and prevention, potentially life-saving, sport event emergency coverage, [41] activity or exercise counselling and basic nutrition advice can be provided through the AT as well [42]. The addition of an AT may increase utilization of preventative services and primary care services by increasing the diversity of skillsets that are now accessible to adolescents.

Post addition of the AT to a clinic in St. Louis, there was approximately a 20% increase in new office visits, 32% increase in the total number of both pre-participation and wellness physicals compared to the previous year. While there are always multiple variables at play, it is still significant to note the increase given that the only change in the clinic environment was the addition of the AT. Addition of an AT aligns with the interprofessional model of the SBHC care delivery system and may enhances the services delivered [65]. The nurse practitioner at the clinic noted that students and athletes realised the variety of services that the clinic offered after having to utilise the AT and the perception of the clinic changed with addition of the AT.

There are limitations regarding the results presented and evaluation of the impact of inclusion of sports medicine health professionals in an SBHC is needed in order to assess its true utility in addressing the aforementioned inequities.

## 3. Conclusions

Inclusion of sports medicine health professionals in an SBHC may be a novel staffing model to deliver comprehensive care that aligns with the national goals, encourages students to visit clinics and increases the safety of students participating interscholastic activities. The positive outcomes with this interprofessional staffing model change do not come without challenges. Buy-in from all the stakeholders is challenging as some of the school staff (teachers, school nurse, coaches) and administrators have never worked with AT or never worked with one in this setting. It represents a culture shift for everyone including the students and athletes and district. Proper education and consistent discourse with all are tantamount to the success of this model and ensure that needs and concerns of all stakeholders are met. It can be advantageous to the entire community that is serviced by SBHC in multiple ways and contribute to the goals of the WSCC to incorporate these health professionals. It can be a novel method of promoting community and individual health initiatives and engaging youth in their health as well. Utilising sports medicine in conjunction with SBHCs to ease the burden on the broader healthcare system is an innovative approach to healthcare policy and adolescent care. Enhancing education through healthcare is a radical policy that could influence multiple drivers and determinants of health.
